# The effect of deficient muscarinic signaling on commonly reported biochemical effects in schizophrenia and convergence with genetic susceptibility loci in explaining symptom dimensions of psychosis

**DOI:** 10.3389/fphar.2014.00277

**Published:** 2014-12-15

**Authors:** Costa Vakalopoulos

**Affiliations:** Richmond Hill Medical CentreMelbourne, VIC, Australia

**Keywords:** PLCB1, neuregulin, DISC1, dysbindin, parvalbumin, cytokines

## Abstract

With the advent of DSM 5 criticism has generally centered on a lack of biological validity of the diagnostic criteria. Part of the problem in describing a nosology of psychosis is the tacit assumption of multiple genetic causes each with an incremental loading on the clinical picture that fails to differentiate a clear underlying pathophysiology of high impact. The aim of this paper is to consolidate a primary theory of deficient muscarinic signaling underlying key clinical features of schizophrenia and its regulation by several important genetic associations including neuregulin, DISC and dysbindin. Secondary reductions in markers for GABAergic function and changes in the levels of interneuron calcium binding proteins parvalbumin and calbindin can be attributed to dysfunctional muscarinic transduction. A parallel association exists for cytokine production. The convergent pathway hypothesis is likewise used to model dopaminergic and glutamatergic theories of schizophrenia. The negative symptom dimension is correlated with dysfunction of Akt and ERK transduction, a major point of convergence. The present paradigm predicts the importance of a recent finding of a deletion in a copy number variant of PLCB1 and its potential use if replicated, as one of the first testable biological markers differentiating schizophrenia from bipolar disorder and further subtyping of schizophrenia into deficit and non-deficit. Potential limitations of PLCB1 as a prospective marker are also discussed.

## INTRODUCTION

A case for hypofunctioning muscarinic transduction is gaining face validity in schizophrenia research. Implicit memory dysfunction and an imbalance of monoaminergic and in particular, dopaminergic neurotransmission due to a primary muscarinic signaling deficit presents a novel paradigm for understanding schizophrenia (Vakalopoulos, 2006). Explicit memory in schizophrenia as tested on recall of paired associates can show performance comparable to controls (Eyler et al., 2008). No unambiguous demonstration of intact implicit memory has been demonstrated (Vakalopoulos, 2011). It is considered a core deficit explained by the theory of muscarinic receptor signaling deficits.

A detailed account of how muscarinic dysfunction could account for symptoms and endophenotypes of schizophrenia has been proposed (Vakalopoulos, 2006). A previous paper showed an imbalance between dopaminergic and cholinergic signaling that could be corrected by neuroleptics (McGeer and McGeer, 1977). They revealed high ChAT (choline acetyltransferase) activities in a number of brain regions suggesting reduced muscarinic receptor signaling. Glutamic acid decarboxylase (GAD) levels were also noted to be low but in four patients they were elevated in certain regions possibly suggesting secondary changes in this enzyme rather than a primary etiology. A goal of this paper is to show how many of the small effect sizes of genetic variation and mutation impact on complex intracellular cascades initiated by occupation of the muscarinic receptor. Mutations or polymorphisms in DISC1, neuregulin and dysbindin can alter the signaling capacity of the muscarinic receptor and thus, facilitate hypersensitivity of dopaminergic transduction.

Innovative studies demonstrating the efficacy of muscarinic agonists in improving core schizophrenic both clinically and in animal models provided a fillip to further exploration of the role of cholinergic neurotransmission beyond the nicotinic receptor (Bymaster et al., 2002). Although M1–M5 subtypes are all implicated in clinicopathological studies, most attention has focussed on M1 and M4 receptors. Animal studies showed that in M4 knockout mice D1 dopamine receptor hyperresponsiveness occurs in the striatum (Gomeza et al., 1999). Several of the authors later propose an indirect mechanism for the pathophysiology of psychosis involving M4 midbrain autoreceptors, a loss which paradoxically increases cholinergic drive of dopaminergic nuclei and neurotransmitter levels (Tzavara et al., 2004). A more direct effect M4 deficit on intracellular signaling and monoamine-muscarinic imbalance was proposed as causal in declarative memory deficits associated with schizophrenia (Vakalopoulos, 2006, 2010).

M1 deficit and monoamine imbalance is a parsimonious explanation of broad domains of schizophrenic symptomatology, including negative symptoms, memory deficits, and psychosis. It is generally accepted that the main target of antipsychotics, both typical and atypical, remains the D2 receptor. There is an inverse modulation of signaling between M1 and D2 receptors suggesting a dominant role for M1R dysfunction in schizophrenia. The relative lack of effectiveness of most agents in cognition and motivation suggests a role for M1 not only as a potential procognitive therapeutic target, but also implicates the pathway in the causal nature of the disorder. Biochemical indices commonly demonstrated in schizophrenia are altered by M1 and to a lesser extent M4 muscarinic receptor modulation. Apart from GAD_67_ these include calcium binding proteins and cytokines.

Finally, it will be argued that genetic variability and discovered pathophysiological changes in schizophrenia support the ongoing debate on distinct deficit and non-deficit subtypes (Strauss et al., 2013; Kirkpatrick, 2014). The stated aim is to provide biological underpinnings for these descriptors where relative muscarinic signaling impairment and corresponding deficits in encoding implicit emotional constructs are allied with avolition and apathy. These are core negative features of the deficit syndrome.

## CONFLUENT PATHWAYS TO AFFECTIVE DYSREGULATION

A biological premise for implicit memory in affective disorders (Vakalopoulos, 2007) was proposed to elucidate the signaling pathways involved in the emotional dysregulation of psychotic disorders (Vakalopoulos, 2010). Convergent signaling of *N*-methyl-D-aspartate (NMDA), brain derived neurotrophic factor (BDNF), dopamine D3R (Gi) and muscarinic M1R (Gq/11) is involved in the learning and expression of emotional behavior. BDNF and its tyrosine kinase receptor have a major role in depression (Eisch et al., 2003). Key elements of the intracellular pathways, ERK1/2 (extracellular signal-regulated kinase) and PI3K/Akt (phosphatidylinositol 3-kinase and protein kinase B) cascades, are implicated in mood regulation and addiction (Einat et al., 2003; Neasta et al., 2011). Consistent with G-protein cross-activation, synergistic effects were noted between Gi-Ras and Gq-PKC-dependent induction of ERK (Blaukat et al., 2000) and is a model for D3-M1 interactive signaling effects. Although, these were independent of tyrosine kinase and PI3K in this study, the muscarinic agonist carbachol activates ERK1/2 via mitogen-activated protein kinase (MAPK) that is protein kinase C (PKC) independent, but Src (tyrosine kinase) and PI3K dependent (Rosenblum et al., 2000). Dopamine D3 receptor (Gi), known to have a role in addictive behavior, interacts with SH3 (Src homolog) domain of growth factor receptor bound protein Grb2 and Src and thus, is likely to signal ERK and Akt activation (Oldenhof et al., 2001).

The phorbol ester PMA in a human breast cancer cell line markedly activates the Raf-MEK (mitogen-activated protein kinase kinase)-ERK but not PI3K-Akt pathway (Moelling et al., 2002). Application of PMA mimics the activity of PKC kinases. However, balanced activation is required for proliferation of cells. Gi/o protein subunits Gβγ signals transactivation of tyrosine kinases and PI3K-Akt pathway. This explains the importance of confluent signals that include not only D3R, but also colocalization of M1/M4 receptors. It leads to the increased production of necessary transcription factors involved in synaptogenesis and the generation of neural networks that underlie memory traces involved in emotional regulation.

The main effects of muscarinic M1 activation are mediated on the one hand, by diacylglycerol (DAG) and inositol triphosphate (IP3), products of PLCβ1 (phospholipase C) activation and on the other by PKC dependent and independent transactivation of growth factor tyrosine kinases. The discovered PLCB1 haplo-insufficiency described below in psychosis would effectively reduce the level of activated PKC isoenzymes and phosphorylated ERK in some brain regions augmenting dopamine signal transduction as in the basal ganglia. The relative integrity of the ERK and Akt pathways could define affective symptom dimensions in schizophrenia differentiating deficit from non-deficit subtypes. Muscarinic sensitization of these pathways would underlie the affective nature of psychosis in bipolar disorder and differentiating it from schizophrenia. The non-deficit subgroup represents an intermediate phenotype between bipolar and negative symptom schizophrenia.

The forced swimming (FST) and tail suspension (TST) tests are animal models of depression that use measures of immobility. This is associated with phosphorylation of ERK 1/2, JNK and p38, members of the MAPK family. Blocking MEK, but also PKC produced anti-depressant like behavior without affecting locomotor activity (Galeotti and Ghelardini, 2012). An increase in platelet membrane-bound PKC activity was found in bipolar disorder patients in response to application of 5-HT (Friedman et al., 1993; Wang et al., 1999). PKC response to acetylcholine (ACh) was not tested. The latter measure would be expected to distinguish bipolar disorder from schizophrenia.

A way of conceptualizing the affective dimension of psychosis proposes a biochemical nomenclature: PLCβ1^-/-^, PLCβ1^-/+^ and PLCβ1^+/+^, where + and – represent normal and deficient allelic or pathway expression to a balanced ERK and Akt activation. Abnormal sensitization of PLCβ1^+^ (affective dimension) where the integrity of the pathway is preserved would involve confluent and dysregulated mechanisms upon the M1 signal. Some support for this thesis comes from a study stimulating muscarinic receptors in human neuroblastoma cells (Grimes and Jope, 1999). Carbachol resulted in a potent increase of neural growth factor (NGF1-A/Egr-1) protein levels and DNA binding activity. This effect was reduced by down regulation of PKC (85%), lower calcium levels (25–35%) and tyrosine kinase inhibitor (35%). Inhibition of MEK1/2 and the mood stabilizer valproate, but not lithium nor carbamazepine, reduce activation by 60%.

### NMDA AND M1 RECEPTOR RESPONSES

Di Chiara et al. (1994) claim that medium spiny striatal neurons are only indirectly affected by non-competitive NMDA antagonists. A primary action on large aspiny cholinergic neurons is proposed (Di Chiara et al., 1994; Vakalopoulos, 2006). The latter are tonically active. Blockade of NMDA receptors on D1 and D2 mediated responses in an experimental model of dopamine deficiency resembles that of scopolamine a muscarinic receptor antagonist (Damsma et al., 1991). Dizocilpine (MK-801), a commonly used non-competitive NMDA antagonist, not only reduces D1-dependent ACh release, but also basal ACh levels. ACh itself enhances striatal NMDA responses via the muscarinic M1 receptor (Calabresi et al., 1998) showing convergence of pathways. It also suggests that the psychosis-inducing effects of NMDA antagonists are mediated through muscarinic inhibition both indirectly, via reduction of the tonic firing of cholinergic striatal neurons and directly, through its effects on muscarinic receptor transduction.

Positive allosteric modulation of M4R reverses MK-801-induced hyperlocomotion and mice model of cognitive deficits (Bubser et al., 2014). NMDA is the excitatory neurotransmitter for large aspiny cholinergic striatal neurons (Vakalopoulos, 2006). Thus, the effects of MK-801 are to a large extent indirectly mediated through striatal cholinergic neurons acting on separate populations that express either M1 or M4 receptors.

Physiological levels of NMDA have been shown to increase both pAkt and pERK (active forms) in an inverted U-type function independent of PKC (Sutton and Chandler, 2002). It involves Gβγ subunit of heterotrimeric Gi/o-protein, PI3K, and calcium. The neuroprotective effects of NMDA require BDNF secretion (Marini et al., 1998). The NMDA antagonist phencyclidine reduces the number of synaptic sites and expression of synaptic proteins by downregulating Trk and these changes are rescued by application of BDNF (Adachi et al., 2013). BDNF also activates pAkt, but is independent of extracellular calcium (Perkinton et al., 2002). Optimally effective memory formation and associated synaptic consolidation probably requires signal convergence of several distinct ligand-receptor couplings.

## PROLIFERATIVE PATHWAYS IN SCHIZOPHRENIA

Excessive or unbalanced functional signaling of Raf-MEK-ERK and PI3K-Akt-mTOR has been strongly implicated in cancer formation (Steelman et al., 2011). mTOR (mammalian or mechanistic target of rapamycin) is a major focal point of convergence of many signaling pathways that lead to protein and lipid synthesis and metabolic regulation (Laplante and Sabatini, 2009). Rates of cancer incidence are significantly reduced in relatives and patients especially prior to first diagnosis of schizophrenia (Ji et al., 2013). This finding is maintained only in males after diagnosis, but overall supports the putative deficient pathway in schizophrenia. In fact, both M1-like and M2-like signaling can phosphorylate Akt and ERK messengers (Kajiya et al., 2012; Wu and Wong, 2006) and is abrogated by regulators of G-protein signaling (RGS; Anger et al., 2007).

A cholinergic autocrine loop for many cancers has been established with muscarinic (M3) agonists increasing and antagonists decreasing tumor growth (Spindel, 2012). However, as in schizophrenic therapeutics muscarinic modulation in cancer remains theoretical and experimental.

### Wnt AND β-CATENIN PATHWAYS

The transcription of the mouse proto-oncogene Wnt (wingless/integration) is secreted as a glycoprotein and interacts with its receptor Frizzled and a coreceptor low density lipoprotein receptor-related protein (LRP5/6). Transcription via the cytoplasmic scaffold protein Disheveled (Dvl) prevents sequestration and thus, degradation of β-catenin by a complex that critically includes glycogen synthase kinase (GSK3; Clevers and Nusse, 2012). The canonical β-catenin and Wnt induction of mTOR regulates stem cell fate (Iglesias-Bartolome and Gutkind, 2011). There are also non-canonical Wnt pathways activating the kinase JNK and calcium signaling and that share a role in neurodevelopment and mature synaptic remodeling (van Amerongen and Nusse, 2009).

The non-canonical pathway upregulates synaptic NMDA receptor currents (Cerpa et al., 2011). Wnt-2 mediated activity-dependent dendritic arborization conversely, depends on NMDA calcium currents, calmodulin-dependent protein kinase activated Ras/MEK/ERK and CREB-dependent transcrption of Wnt 2 (Wayman et al., 2006). Synaptogenesis associated increases in dendritic spine density is also stimulated by BDNF induction of CREB transcrition (Lesiak et al., 2013). Tyrosine receptor kinases facilitate activation of the canonical Wnt/β-catenin pathway via ERK/LRP6 (Krejci et al., 2012). Another study using rat hippocampal slices showed that late phase long-term potentiation (LTP) under weak synaptic stimulation can be achieved only in the presence of the β-agonist isoproterenol (Ma et al., 2011). Akt inactivates GSK3 by phosphorylation, in turn activating mTOR, which also requires Wnt signaling. NMDA antagonist APV-5 blocks LTP and abolishes Wnt3a-mediated nuclear localization of β-catenin (Chen et al., 2006).

M1 muscarinic agonists likely enhance Wnt pathways by at least, a PKC mediated inactivation of GSK3 (Inestrosa and Toledo, 2008). It has in fact been shown that M1 muscarinic receptor and the growth factor receptor (EGFR) mediate proliferation of progenitor cells in salivary gland (Knox et al., 2010). Gαq mediates M3 muscarinic reduction in GSKβ activity and increase in β-catenin accumulation (Salmanian et al., 2010).

In mouse models of learning Wnt signaling in the amygdala is necessary for late consolidation of a fear-conditioning task where initial acquisition is unimpaired by local application of Wnt1 or its inhibitor Dickkopf-1 (Dkk-1; Maguschak and Ressler, 2011). Initial decrease in β-catenin destabilizes synapses allowing modification by reduced binding to the cell adhesion molecule cadherin with a subsequent restoration of the complex for consolidation. BDNF increases synaptogenesis by also disrupting these complexes presynaptically (Bamji et al., 2006). Mice with Dkk-1 infused into the dorsal hippocampus showed no distinction in exploring a novel object from a familiar one introduced 24 h earlier suggesting loss in object recognition (Fortress et al., 2013). This was associated with a significant decrease in phospo-GSK3β, its inactive form.

There is thus, a complex interplay between various receptors and their signaling cascades and subtle differences in transduction that might result from a dynamic balance of monoamine-muscarinic cholinergic coactivation of neuronal synapses could signal a modified course of Hebbian plasticity. This means that varying the monoamine-muscarinic signal is categorical for cognition and flexible behavior. Thus, a putative dual model of hierarchical control of signal transduction is represented at a molecular level by enhancement of single synaptic events or dendritic formation associated with reinforcement to remolding of networks and attrition of synapses in the context of new contingencies that require adaptive change.

### Wnt AND SCHIZOPHRENIA

Schizophrenia is regarded as a neurodevelopmental disorder, however, it is unclear whether the Wnt signaling cascade itself directly contributes to risk (Panaccione et al., 2013). A study that looked at peripheral blood Wnt biomarkers of 19 patients with a DSM-IV diagnosis of psychosis and using the scales for the assessment of positive (SAPS) and negative (SANS) symptoms found that SANS, but not SAPS was correlated with several factors (Bousman et al., 2013). A negative correlation was significant for Dvl, trending for a transcription factor in the canonical pathway (TCF4) and a positive correlation approaching significance for GSK3. Dvl1 knockout mice exhibit reduced social behaviors and sensorimotor gating as measured by prepulse inhibition of acoustic and tactile startle responses (Lijam et al., 1997).

**FIGURE 1:**
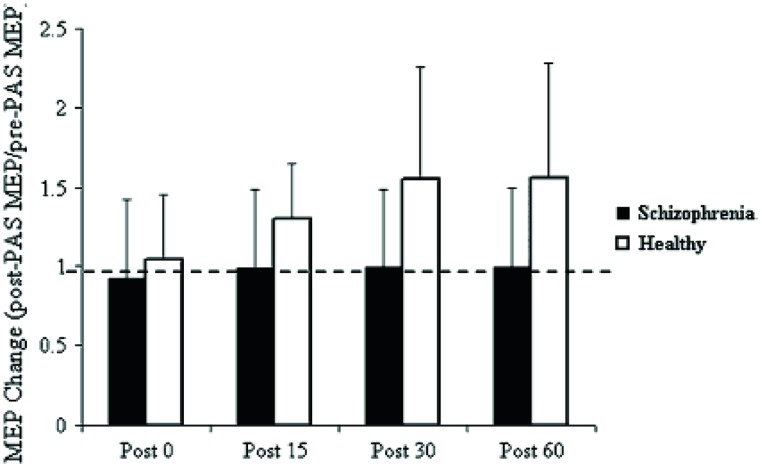
**Effect of PAS on MEP amplitude.** Values represent a ratio of post-/pre-MEP amplitude. Values greater than 1 (dashed line) represent a PAS-induced MEP facilitation. Results demonstrate that patients with SCZ demonstrated no MEP facilitation (Frantseva et al., 2008).

**FIGURE 2:**
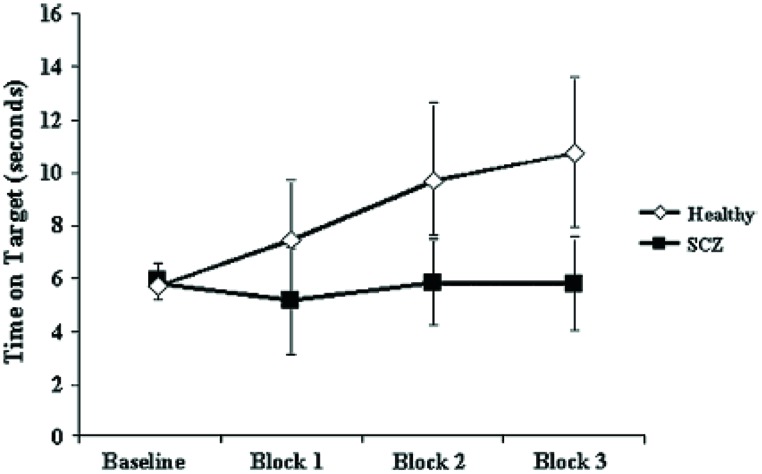
**Impaired motor skill learning in SCZ.** Data represent mean ± standard deviation acquired for each of the three blocks of data acquired. Motor skill learning was derived through the rotary pursuit task. In this task, participants are required to hold a stylus upon a flat surface under which the target rotated and follow the target as it moved about its path. A photoelectric device measured the time (in seconds) that the stylus was held correctly over the target for each trial. Each block of skill learning consisted of eight 20-s trials with a 20-s intertrial interval. These data suggests that unlike healthy subjects, patients with SCZ failed to demonstrate significant motor learning (Frantseva et al., 2008).

Pathognomonic learning impairments have been proposed for schizophrenia and these should reflect signaling changes. It remains important to describe these correlations in higher-level psychological terms (Vakalopoulos, 2010). An elegant study in a group of 15 patients with a DSM IV diagnosis of schizophrenia or schizoaffective disorder showed a profound deficit in LTP-like enhancement of motor evoked potential (MEP) amplitude during a paired associative stimulation paradigm (**Figure 1**). The experiment involved pairing of median nerve electrical stimulation with transcranial electrical stimulation TMS of the corresponding contralateral motor area (Frantseva et al., 2008). The same patients demonstrated no learning on a rotary pursuit task, a measure of skill learning (**Figure 2**). Patients failed to improve their ability to stay on target for longer periods of time compared to controls.

The graphs suggest a specific and fundamental principle of cognitive impairment in schizophrenia that has not been sufficiently appreciated. Most reaction time experiments in this group show improvement in spite of overall and maintained marked slowing in performance compared to normal subjects. This is taken, unreasonably, to show skill learning, i.e., in the non-declarative sense. However, the current results clearly demonstrate a core deficit in this type of learning. Rotary pursuit, as tested in this study, can be taken as a relatively pure measure of procedural learning where overt strategy contributes little to improved time on target, whereas improvement in conscious control can index slow RT improvements in other tasks.

Further, a special relationship is suggested between specific aberrations in intracellular signaling and the negative symptom dimension in schizophrenia, which is manifested as synonymous impairments in procedural or skilled acquisition or more broadly, implicit memory.

## GENE DELETIONS AFFECT MUSCARINIC SIGNALING IN PSYCHOSIS

**FIGURE 3:**
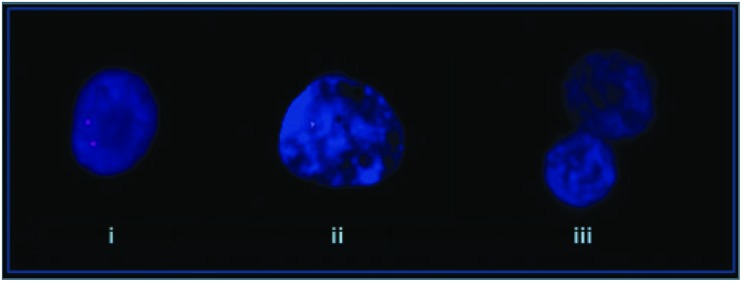
**Molecular cytogenetic analyses of normal control and schizophrenia affected patients.** (i) Normal control, (ii) mono-allelic deletion, and (iii) bi-allelic deletion (Lo Vasco et al., 2012).

A study identified a deletion of the PLCB1 gene (**Figure 3**) in the orbito-frontal cortex for 4 of 15 post-mortem schizophrenic patients (Lo Vasco et al., 2012). The PLCβ signaling pathway transduces Gq/11. In a mouse model of PLCβ1 deletion selective attenuation of muscarinic M1 and not serotonin-induced 5-HT 2A/2C phosphoinositide hydrolysis was demonstrated (Kim et al., 2007), suggesting specificity to this signaling pathway. Microarray studies have shown reduced mRNA for PLCβ1 in the dorsolateral prefrontal cortex (DLPFC) of schizophrenic patients. Widespread cortical muscarinic receptor decreases in a subset of probands has already established a precedent for an index of dysfunction along this pathway (Gibbons et al., 2013). The significance of muscarinic receptor deficits now appears clearer as a secondary change to genetic events like PLCB1 microdeletions. PLCB1 KO mice display typical endophenotypes for schizophrenia and reduced levels of M1 receptor binding (McOmish et al., 2008).

In three of the four positive cases reported by Lo Vasco et al. (2012) a single allele deletion was present in practically all nuclei analyzed. In two of these the mode of death was suicide. In the other patient both alleles were deleted in 76% of nuclei and a single allele in the remaining 24%. This patient did not commit suicide. The low numbers and lack of clinical descriptors make it difficult to draw any firm conclusions, but monoallelic versus deletion of both alleles are a potential concomitant of a non-deficit/deficit dichotomy, respectively. In another study on patients with major depressive disorder no such deletions of the PLCB1 gene were discovered (Lo Vasco et al., 2012) highlighting the distinction in nosology between affective and psychotic illness. 26% of the schizophrenic sample demonstrated deletions of the PLCB1 allele, but none of the normal controls and this figure may prove to be higher with the use of smaller probes. A study of high-density Irish families for psychotic illness demonstrated a linkage to chromosome 20p close to the locus for PLCB1 (Fanous et al., 2008). Deficit syndrome had the highest LOD score of any latent class for the genome.

In a post-mortem brain study of 15 bipolar subjects bipolar disorder (BPD) a single deletion of PLCB1 was discovered in only one sample (Lo Vasco et al., 2013). The association of PLCB1 deletion and bipolar disorder appears much weaker assuming a valid initial diagnosis of BPD. Alternatively, based on the predictive strength of the theory of a muscarinic transduction deficit, a biological marker could determine unambiguously the nosological category of a case of psychosis and the positive bipolar post-mortem case in this instance would be confidently reclassified as schizophrenic. A distinction was made in subgroups of psychotic disorder when ascertaining phosphorylation of Akt (pAkt) by neuregulin-1 (NRG-1) stimulation of ErbB receptor in B lymphoblasts (Kéri et al., 2009). The ratio pAkt/Akt was significantly reduced in schizophrenia, but those with bipolar or major depressive psychosis did not differ from controls. All psychotic subgroups were similarly impaired, highlighting the nosological specificity of the signaling pathway.

A further analysis of the categorical significance of single versus double deletions of PLCB1 alleles is warranted. A partial dysfunction of the M1 signal augments catecholaminergic transduction. None the less, there is some conservation of the convergent M1-NMDA signal on Ras-ERK and PI3K-Akt intracellular pathways involved in affective dysregulation. Thus, the high suicide rate in single allele deletion. No such behavioral association would be expected with double allelic deletions, i.e., a more profound disruption of Akt and ERK pathways and a putative predictor of deficit syndrome.

## THE PARADOX OF RELATIVE VERSUS ABSOLUTE DEFICITS

The concept of convergent signaling states that the primary deficit in the muscarinic receptor and its transduction products creates a relative imbalance of monoaminergic cholinergic internal states of a neuron. In attention deficit hyperactivity disorder ADHD recent studies demonstrated a 50% reduction in muscarinic receptor binding (Coccini et al., 2009; Johansson et al., 2013). However, it has been proposed that a defective catecholaminergic response unmasks cholinergic muscarinic mediated prepotent behaviors (Vakalopoulos, 2007). This suggests the muscarinic findings are secondary. An explanatory model comes from an altogether different body system, the pulmonary airways. Respectively β2-adrenergic (β2AR) and muscarinic M3 agonism produce bronchodilation and bronchoconstriction and this classic understanding of antagonist effects is the basis of asthma therapeutics. In contrast to its bronchodilator effect chronic use of β-agonists leads to a sensitized mechanism of brochoconstriction. Transgenic mice either lacking β1 and β2 receptors (βAR^-/-^) or overexpressing βARs in airway smooth muscle caused a reduction of or enhanced bronchoconstriction, respectively (McGraw et al., 2003). Notably, PLCβ1 protein level is reduced by 60% in βAR^-/-^ mice, but is at twice the basal level in mice overexpressing the receptors. Thus, adrenergic stimulation modulates protein expression of a key muscarinic signaling pathway. Deletion of the PLCB gene was previously described to be associated with muscarinic receptor reduction and may explain the ADHD findings of absolute reduction but putative relative increase in the muscarinic signal.

## GENE MARKERS OF RISK PATHWAYS IN SCHIZOPHRENIA

A model of hypomuscarinic transduction offers a heuristic approach to multifactorial genetic associations that converge on this pathway. A good example of this is the candidate gene for schizophrenia RGS4 (regulator of G-protein signaling). RGS4 directly modulates Gq/11 and Gi subclasses of Gα subunits, but not Gs (Druey et al., 1998). Further, this negative regulator of G-protein signaling directly interacts with PLCβ1 (Dowal et al., 2001).

DISC1, neuregulin-1 (NRG-1) and dysbindin are commonly implicated genetic risk loci of the disease. These gene transcripts have complex effects in neurodevelopment and adult synaptic plasticity, and are directly involved in signaling cascades implicated in disease generation. DISC1 and neuregulin 1 transcend the traditional dichotomy in psychiatric diagnosis, demonstrating susceptibility for an affective spectrum of psychosis from schizophrenia to bipolar disorder and even major depression (St. Clair et al., 1990; Green et al., 2005; Balu and Coyle, 2011). NRG-1 is also associated with a non-deficit subtype of schizophrenia.

A knockdown of DISC1 in newborn neurons of the adult brain does increase Akt activity (Kim et al., 2009). NRG-1 activates MAPK (ERK) and PI3K via ErbB4 tyrosine kinase receptor and both NRG-1 and ErbB4 are elevated in the prefrontal cortex of schizophrenic patients (Chong et al., 2008) or at least enhanced transduction is noted in post-mortem brains (Hahn et al., 2006). The ErbB4 risk haplotype for schizophrenia is associated with increased levels of transcript for the catalytic subunit of PI3K, p110δ (Law et al., 2012). This in fact, reduces levels of PI(3,4,5)P3 and presumably phosphorylated Akt. The effects are specific to the PI3K pathway because no genetic effects of the ErbB4 risk haplotype were found on ERK-MAPK signaling. This and the fact that PKC only weakly activates PI3K-Akt and high Akt activity suppresses ERK (Moelling et al., 2002) suggests selective dysfunction of closely related pathways and is of putative relevance to dissociation of the negative symptom dimension in psychosis. The effects of DISC1 and neuregulin 1 on ERK and Akt pathways explain, at least partly, the prominence of associated mood disorders. In other words, the manifestation of mood symptoms depends on the relative preservation of signal transduction involving Akt and or ERK.

Dysbindin is reduced in the prefrontal cortex of patients (Weickert et al., 2004). Downregulation of dysbindin expression suppresses PI3K pathway and Akt phosphorylation levels (Numakawa et al., 2004). Several studies have demonstrated that psychotic individuals with a high-risk haplotype of dysbindin may be associated with more pronounced negative symptoms (Fanous et al., 2005; DeRosse et al., 2006; Corvin et al., 2008).

These same genetic markers also have effects on neurotransmitter imbalance potentiating monoaminergic transduction. DISC1 is associated with reduced cytosolic PDE4 impairing breakdown of cAMP and raised NRG-1 reduces NMDA efficiency (Hahn et al., 2006). Mutant astrocyte DISC1 in mouse models reduces NMDA transmission by decreasing D-serine levels (Ma et al., 2013)

## NEUREGULIN-1: A MODEL CONVERGENCE OF GENETIC RISK

Phenotypic expression of NRG-1 mutations in animal models implicate dysregulation of the muscarinic receptor and an imbalance of catecholaminergic effector response. Dilated pupils in a NRG-1 mouse mutant did not respond to the non-specific muscarinic agonist pilocarpine and showed a concomitant reduction of the muscarinic M3 receptor in the sphincter pupillae (Chen et al., 2011). This partial loss of function mutant induced a decrease in EGF-type neuregulin isoforms. Treatment of isolated chick atrial myocytes with NRG-1 (EGF-like domain) also induced a decrease in muscarinic M4 receptor mRNA and carabachol (cholinergic agonist)-evoked outward rectifying currents (Ford et al., 2003). Cardiomyocytes were treated with the β-adrenergic agonist isoproterenol and the antagonizing response to carbachol was tested in wild-type and mice heterozygous for NRG-1 deletion (Okoshi et al., 2004). Although M2R and Gi/o protein levels were normal in heterozygous mice, carbachol failed to attenuate raised intracellular calcium and contractile responses. The anti-adrenergic effects of NRG-1 itself are blocked by the muscarinic receptor antagonist atropine (Lemmens et al., 2005).

## DISC1 AND VENs

**FIGURE 4:**
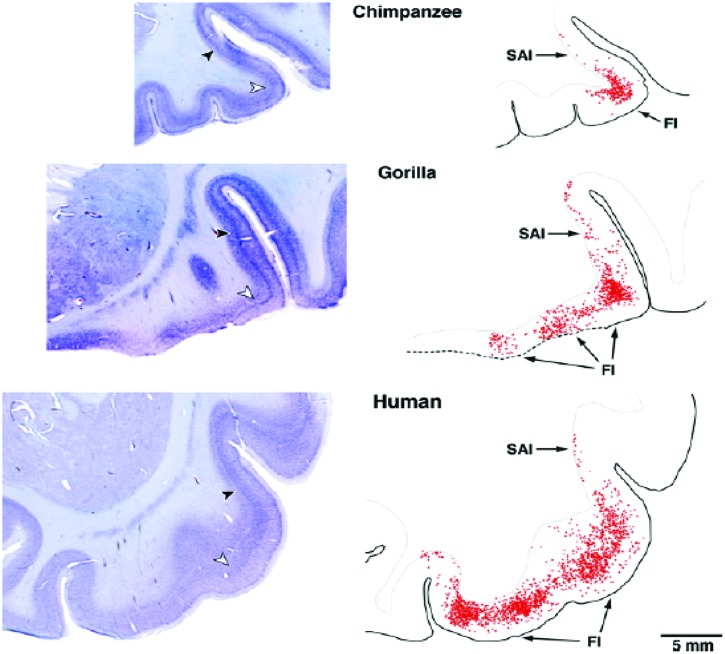
**von Economo neurons are present in Layer V of primate insula cortex (Allman et al., 2010)**.

**FIGURE 5:**
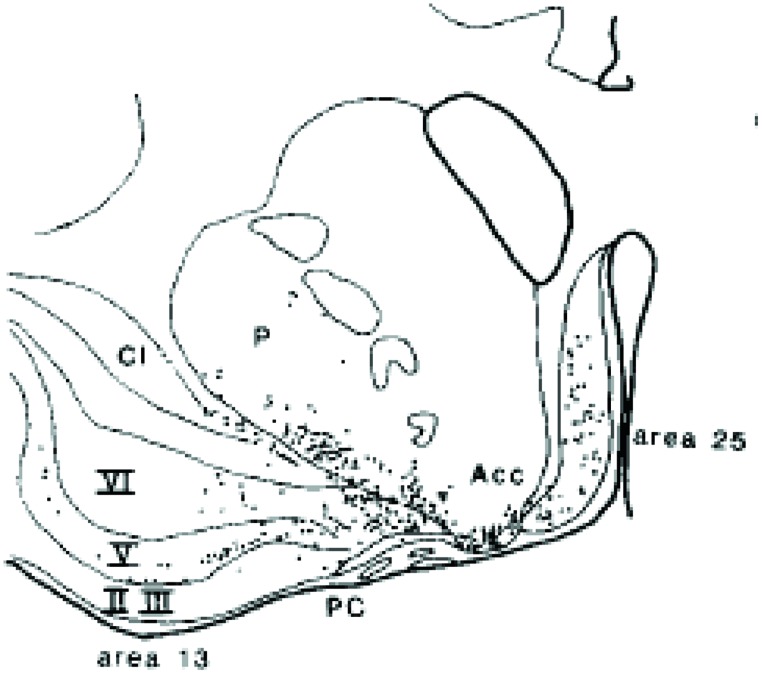
**Projection neurons from the insula deep layers to the cholinergic basal forebrain (Russchen et al., 1985)**.

von Economo neurons (VENs) are large spindle-shaped neurons in layer V of predominantly the frontoinsular (FI) cortex and more irregularly in the anterior cingulate cortex (**Figure 4**). They possess single basal and apical dendrites with restricted branching that span most layers (Watson et al., 2006). The locations of VENs overlap with neurons that project to the nucleus Basalis of Meynert from the anterior granular insula (**Figure 5**). They could specifically serve cholinergic corticopetal activation, a function proposed for the agranular insula in implicit memory encoding (Vakalopoulos, 2010, 2013).

Ninety percentage of VENs are DISC1 positive, while only 37% of other layer V neurons are likewise positive (Allman et al., 2010). That they are potentially important in neuropsychiatric disease is suggested by the finding of a significant reduction in density of VENs in the anterior cingulate cortex inversely related to duration of illness in schizophrenia, but not bipolar subjects (Brüne et al., 2010). Mutations of DISC1 gene would be expected to alter the output of VENs and inducing a relative hypocholinergic state.

### bvFTD

The behavioral variant of frontotemporal dementia (bvFTD) is characterized by insidious changes in personality including social withdrawal, apathy and emotional blunting, but also marked stereotypies (Harciarek et al., 2013; Pose et al., 2013). The clinical profile overlaps most notably with negative symptoms of schizophrenia and is consistent with deficient cortical cholinergic input to cortex. This is presumably mediated through cortical M1R. Although, the positive symptoms of psychosis are relatively low in FTD (up to 13.3%), those patients with specific genetic mutations can have much higher rates (up to 40%; Piguet and Hodges, 2013). One of the most characteristic post-mortem findings in bvFTD is frontoinsular degeneration with loss of over 50% of VENs and fork cells (Kim et al., 2012).

Diagnoses of schizophrenia, schizoaffective, and bipolar disorders can be made early in the course of bvFTD (Velakoulis et al., 2009). Also schizophrenia-related psychosis is observed significantly more frequently in family members of FTD probands than those with Alzheimer’s disease (Schoder et al., 2010). The acetylcholinesterase inhibitor rivastigmine mitigated behavioral changes as measured by the Neuropsychiatric Inventory including psychotic symptoms in an open label study of FTD (Moretti et al., 2004). Treatment of FTD with antipsychotics is generally regarded as ineffective. However, in another study donepezil did worsen clinical state (Mendez et al., 2007). The contradiction may be explained by the finding that donepezil has antagonistic properties at muscarinic receptors (Ago et al., 2011).

## GAD_67_, INTERNEURON MARKERS AND THE M1 RECEPTOR

The 67 kDa isoform of the glutamate decarboxylate mRNA, a GABA synthesizer, is reduced in the DLPFC of schizophrenic subjects in the absence of obvious cell loss. The study included non-medicated patients (Akbarian et al., 1995). 50% of DLPFC parvalbumin (PV) mRNA+ neurons in this group lack detectable levels of GAD_67_ mRNA compared to only 10% in normal controls (Hashimoto et al., 2003). Levels of PV, a calcium-binding protein are also reduced, although PV-expressing cells may be normal in number (Lewis et al., 2008). Overall, 25–35% of GABA neurons lack the transcript for GAD_67_ mRNA in schizophrenic cohorts. Reductions are not specific to PV neurons, but also involve mRNA for interneurons expressing somatostatin (SST)/neuropeptide Y (NPY) and for the GABA membrane transporter, GAT-1. These changes in schizophrenia extend to other cortical areas including anterior cingulate, primary motor and primary visual regions (Hashimoto et al., 2008). GAT-1 regulates reuptake of synaptically released GABA. Using magnetic resonance spectroscopy (MRS) centered on bilateral calcarine sulci, a study found a significant 10% decrease in GABA levels in schizophrenic subjects (Yoon et al., 2010).

Up to half of SST+ and 80% of PV+ interneurons express tropomyosin-related kinase (TrkB; Gorba and Wahle, 1999), a tyrosine kinase receptor for BDNF. TrkB and BDNF mRNA transcripts are reduced in DLPFC schizophrenic patients and a significant correlation exists between TrkB, but not BDNF and GAD_67_ (Hashimoto et al., 2005; Weickert et al., 2005). Calretinin immunopositive neurons appear not to be affected and do not express TrkB.

It was suggested that deficient neurotrophin signaling is an upstream causal event in decreased expression of GABA markers (Lewis et al., 2005). ErbB4, the tyrosine kinase receptor for neuregulin-1, is expressed exclusively on GABAergic interneurons and 50% of these are PV-IR (immunoreactive; Fisahn et al., 2009; Buonanno, 2010). The cytoplasmic tails of ErbB4 receptor and NMDA N2A and N2B subunits interact through the post-synaptic density protein (PSD-95; Garcia et al., 2000). PSD-95 is a member of the membrane-associated guanylate cyclases (MAGUKs) involved in signal transduction. The non-specific muscarinic antagonist scopolamine reduces the expression of BDNF, but also neuronal cell markers including PSD-95 (Konar et al., 2011). Repetitive animal exposure to the NMDA antagonist ketamine causes a reduction in the expression of GAD_67_ and PV (Behrens et al., 2007).

In striate visual cortex (V1) of the macaque monkey PV-IR neurons constitute ∼75% of inhibitory neurons (Disney and Aoki, 2008). 87% of these express M1 muscarinic receptor, compared with only 40% of CR cells. M2 muscarinic receptors are expressed in 32% of PV+ cells suggesting substantial but limited overlap in M1 and M2-expressing cells. Vakalopoulos (2006) considers colocalization to be governed by a dominant subtype, M1- or M2-type for any particular group of neurons. Parallel to PV findings in macaque V1, 93% of rat striatal neurons expressing SST were labeled by a probe for M1R, but only 15% by M4 probe (Bernard et al., 1992). Thus, M1R deficient signaling in schizophrenia can explain reductions in BDNF, GAD and calcium binding protein markers.

Interleukin-4 (IL-4) applied to retinal rat cell cultures increased uptake of [^3^H]-GABA in a dose-dependent manner and was blocked by an inhibitor of the GAT-1 transporter (Sholl-Franco et al., 2002). This effect was dependent on M1R, protein kinase C (PKC), intracellular calcium levels and a tyrosine kinase receptor. A 90% increase in GAD_67_ expression was also induced by IL-4. The *klotho* mutant strain of mice demonstrate reduced phosphorylation of hippocampal janus kinase 2 (JAK2) and signal transducer and activator of transcription (STAT3; Park et al., 2013). This induces a reduction in several cholinergic parameters and a selective loss in protein expression and binding density of M1 muscarinic receptor and a concomitant decrease in PKCBII, p-ERK, p-CREB, and BDNF. NMDA-dependent LTP is also impaired. All transcript reductions and LTP are reversed by the purported M1R agonist, McN-A-343. The normalizing effects of McN-A-343 are in turn reversed by a BDNF receptor tyrosine kinase inhibitor suggesting mediation of the muscarinic agonist effects. However, non-selectivity and complex effects have been reported for McN-A-343 including M4R agonism and serotonergic effects.

Although, GABA related interneuron findings are amongst the most robust in post-mortem schizophrenic samples they are also present to a variable degree in mood disorders. PV is reduced in Brodmann area 9 of DLPFC of BPD and SST in major depressive disorder (MDD), but not GAD_67_ (Sibille et al., 2011). However, GAD_67_ is reduced in the orbitofrontal cortex (OFC) and CA4 region of the hippocampus in non-psychotic MDD and TrkB in CA4 region of BPD (Thompson et al., 2009, 2011). GAD_67_ is also down in the OFC of BPD. This suggests that GAD_67_ does not directly contribute to the psychotic phenotype. The overlap of deficient GABA markers may result from a common reduction in neurotrophic BDNF protein density (Dunham et al., 2009).

### THE SPECIAL CASE OF CALBINDIN

**FIGURE 6:**
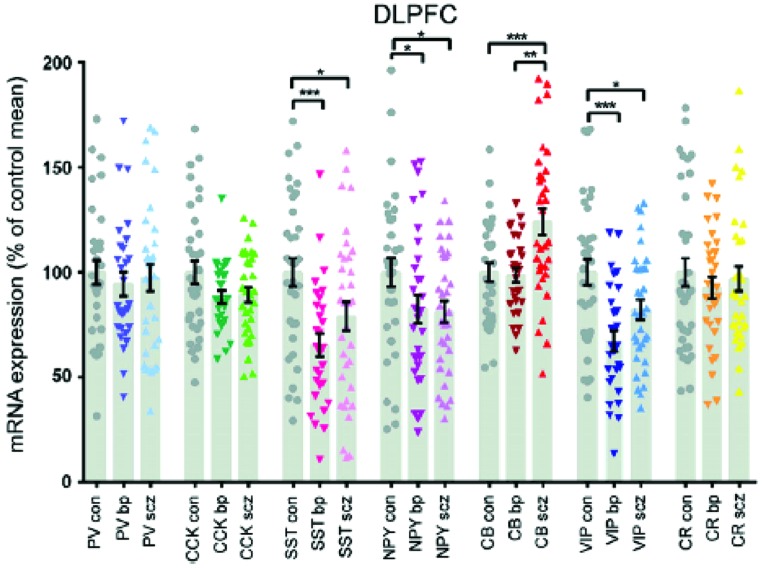
**The interneuron protein marker calbindin is elevated in schizophrenia, but bipolar subjects do not differ from controls (Fung et al., 2014)**.

The density of calbindin immunoreactive neurons CB-IR in the prefrontal cortex, areas 9 and 46, of a small number of schizophrenics was 50–70% greater than matched controls (Daviss and Lewis, 1995). The reactivity was largely from interneurons of the superficial layers and the magnitude of the increase would suggest change in neuronal number. A non-significant increase in calretenin-IR was also found. This was an immunohistochemical study and other similar studies showed no (Tooney and Chahl, 2004) or even decreased CB-IR (Beasley et al., 2002). However, a study looking at mRNA transcripts of calcium binding proteins CBP showed a 14.9% rise in CB, while most other interneuron markers were reduced including PV, somatostatin, cholecystokinin, vasoactive intestinal protein VIP and neuropeptide Y (Fung et al., 2010). The same research group replicated an increase of 22.7% in CB of a separate cohort (Fung et al., 2014). Although they reported decreases in somatostatin and VIP mRNA in the prefrontal cortices of both schizophrenic and bipolar disorder subjects, only CB discriminated between them. There was a relative increase of 22.5% in CB in schizophrenics and the level in bipolar disorder did not differ from controls (**Figure 6**). The reason for the discrepancies between some of the studies is not entirely clear, but methodology or subgroup selection can have profound effects on results. Further, phenotypic expression does not always match changes in gene transcription.

Raised calbindin IR and mRNA are a significant deviation from more general reductions in interneuron markers. This suggests differences in neuromodulatory effects. 60% of CB-IR neurons (12% of the GABAergic population) express M1 muscarinic receptors (Disney and Aoki, 2008). In an animal model of PLCB1 deficiency homozygous PLCB1 knockout mice showed a 3-fold increase in the number and density of hippocampal granule cells compared to wild-type litter-mates (Manning et al., 2012). Phenotypic analysis with confocal microscopy showed these to be mature cells expressing CB. No other relevant markers were assessed, but the data are consistent with the selective increase in CB being related to M1R transduction deficits.

Schizophrenia but not bipolar nor controls showed an increased expression of NR2A mRNA by calbindin-containing interneurons of the anterior cingulate cortex (Woo et al., 2008). The same group demonstrated undetectable levels of NR2A mRNA co-expressed in up to 73% of GABAergic interneurons of both schizophrenia and bipolar subjects (Woo et al., 2004). Although the former study did not reveal overall changes in calbindin mRNA, it does suggest a selective proliferative capacity of CB-IR neurons in schizophrenia. Long-term scopolamine use in rats reduces the NMDA subunit NR2A protein levels in the anterior cingulate cortex confirming a muscarinic interaction (Doguc et al., 2012). The NMDA antagonist MK-801 in perinatal mice caused an increase in CB and reduction in calretinin mRNA as well as reduced PV immunoreactivity in the medial prefrontal cortex mPFC (Gilabert-Juan et al., 2013). A point mutation of the DISC1 gene in mice also resulted in reduced PV-IR neurons of the mPFC and increased CB-IR in the DLPFC compared to wild-type (Lee et al., 2013).

## CYTOKINES

Abnormal regulation of inflammatory markers in schizophrenia is considered by some investigators as pointing to a possible causal etiology. Alternatively, putative transduction deficits by neuromodulators of peripheral blood mononuclear cells could be an incidental window into core disturbances within the central nervous system.

*In vitro* stimulation of interleukin-2 (IL-2) production by T lymphocytes is clearly deficient in a subset of schizophrenic patients (Villemain et al., 1989). More than half of the patients in this study did not show reactivity above baseline including all three patients with undifferentiated subtype. This has been replicated in both treated and untreated patients and in further *in vitro* and *in vivo* studies consistent with the status of a trait marker (Bessler et al., 1995; Arolt et al., 2000; Asevedo et al., 2014). A meta-analysis of cytokines in schizophrenia also found a consistent elevation of interleukin-6 (IL-6; Potvin et al., 2008).

Positive and depressive symptoms were unrelated to IL-2 levels, but a negative correlation was described between IL-2 and negative symptoms and positive correlation with age of onset (Ganguli et al., 1995; Asevedo et al., 2014). Further, IL-6 levels were significantly higher in deficit versus non-deficit patients that were antipsychotic naïve and the latter did not differ from controls (Garcia-Rizo et al., 2012).

### NEUROMODULATION OF CYTOKINE PRODUCTION

Acetylcholine appears to function as an autacoid in T-cell mediated immunity. ACh enhances mitogen-activated IL-2 production and is mediated by MAPK/ERK signaling (Okuma and Nomura, 2001; Nomura et al., 2003). Conversely, ACh attenuates release of IL-6 and is probably mediated by the muscarinic M4 receptor (Borovikova et al., 2000; Zhang et al., 2013). Cholinergic muscarinic effects are reciprocal to those of β-adrenergic stimulation, which enhances IL-6 and inhibits IL-2 production by activating an adenylyl cyclase cAMP cascade (Kammer, 1988; Papanicolaou et al., 1998). Akt 1 levels were significantly reduced in schizophrenia (Emamian et al., 2004; van Beveren et al., 2012). Akt 1 converges with neurotrophin-associated pathways in immune regulation including IL-2 signaling. The inverse relationship between catecholamines and muscarinic receptor function is analogous to findings in the brain and the striatum in particular.

### CYTOKINES AND A NOSOLOGY OF PSYCHOSIS

**FIGURE 7:**
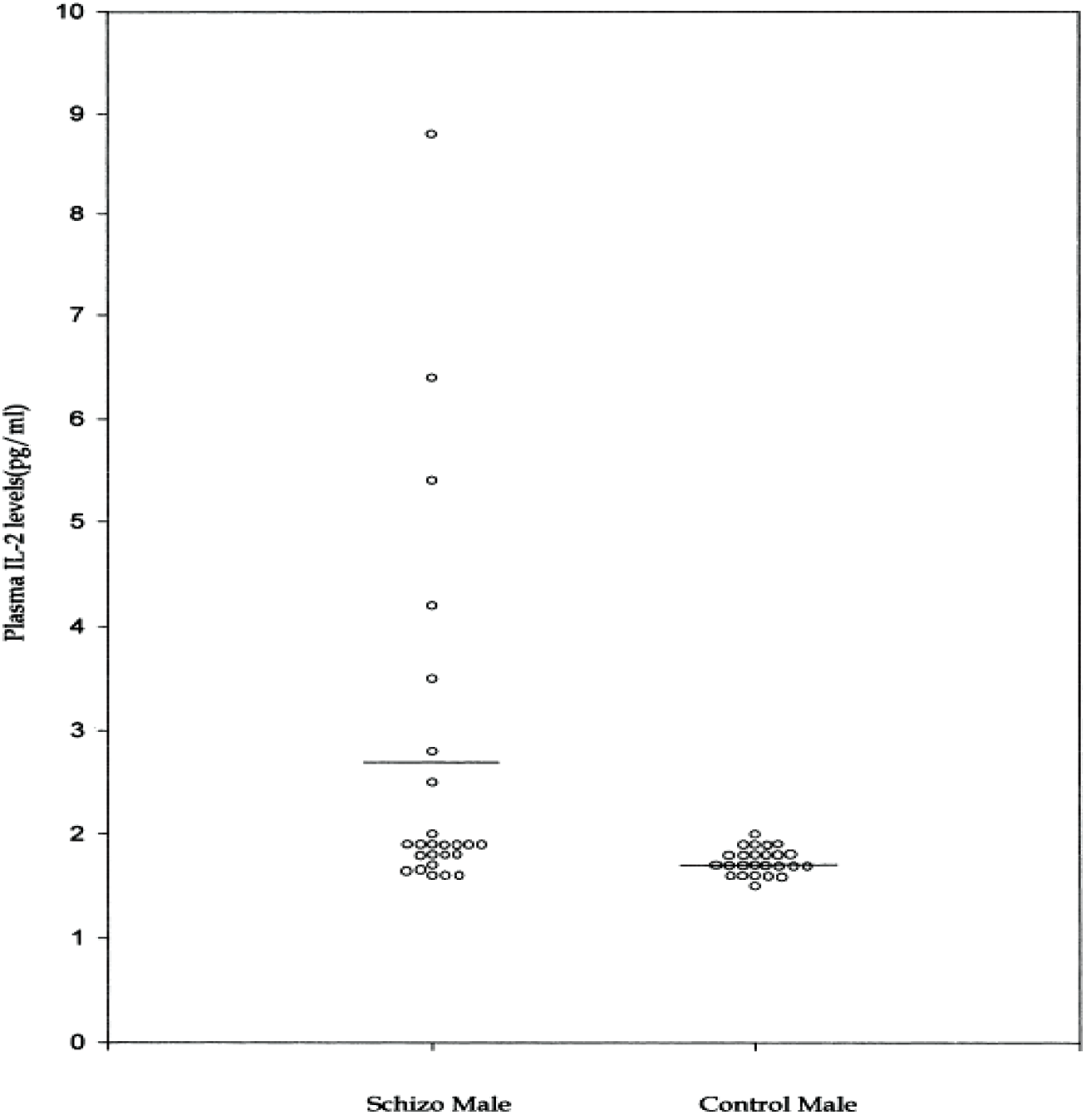
**Plasma IL-2 levels in male schizophrenic patients and normal controls (Kim et al., 2000).** Notice two subgroups.

**FIGURE 8:**
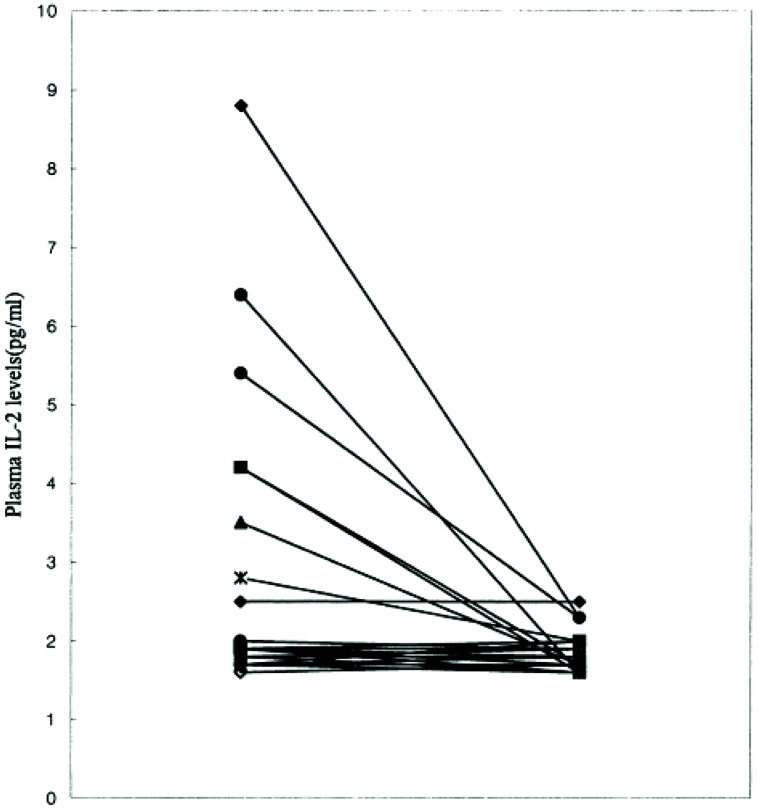
**Iinterleukin-2 levels normalize after 8 weeks of treatment in schizophrenic patients (Kim et al., 2000)**.

As is common with other endophenotypes not all studies are in agreement with the above findings. There is not only interstudy, but also intrastudy variability as would be expected from the study of heterogeneous populations. No difference was found in IL-2 levels between schizophrenic subjects and controls in one study of 16 paranoid types, 10 of which had a first onset presentation (Gattaz et al., 1992). The plasma level of IL-2 was significantly higher in a study where it correlated positively with the SAPS (Kim et al., 2000). Although, IL-6 level did not differ from controls, it did correlate positively with the SANS and the duration of illness. The cohort consisted of 14 paranoid and 11 undifferentiated subgroups. It remains plausible that the former group drove the mean elevation in IL-2 levels and the latter IL-6. This is based on the finding that only six patients were outside the control range for IL-2 (**Figure 7**) and the measures moderated to the normal range after 8 weeks of treatment (**Figure 8**). The same research group showed higher serum levels of IL-2, but lower mitogen-induced production of IL-2 in schizophrenic subjects compared to controls (Kim et al., 1998). A similar argument could be invoked to explain the differential findings in this group of nine paranoid type and seven undifferentiated patients. The associations were not looked at in the study presumably because of low statistical power, but would be of some interest.

The data suggests that the majority of cases of paranoid schizophrenia might better be grouped under a spectrum of affective psychoses where IL-2 is a state-dependent marker. Thus by analogy, in one study of bipolar subjects levels of IL-2 and IL-6 were increased in the manic state compared to controls, but only IL-6 remained elevated in the depressive phase (Brietzke et al., 2009). Mood symptoms were positively correlated with the cytokines. A meta-analysis showed increased IL-6 in mania, normalizing in the euthymic state and a trend for increases in IL-2 only in studies that used stimulation (Modabbernia et al., 2013). Premedicated manic patients had non-significantly higher serum IL-2, which became significant upon remission (Liu et al., 2004). It is not clear why IL-2 levels were low in another anomalous study of both manic and depressive bipolar subjects (Ortiz-Dominguez et al., 2007).

## CONCLUSION

**FIGURE 9:**
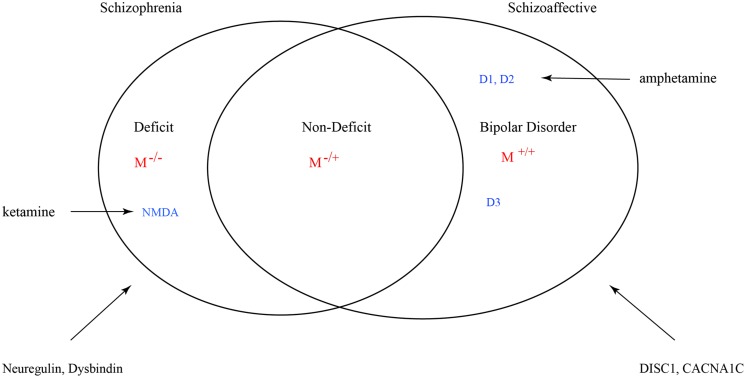
**Venn diagram for a nosology of psychosis.** M represents the relative muscarinic deficit and correlates with primary negative symptoms. D, dopamine receptor subtypes. D3 sensitizes M receptor function. Across the spectrum dopamine-muscarinic imbalance results in an overlapping phenotype in positive symptoms.

Many factors can influence the balance between monoaminergic and muscarinic cholinergic cascades such that variance is exhibited in the full phenotype of psychosis. However, genetic mutations such as copy number variants (CNVs) and single nucleotide polymorphisms (SNPs) that result in a muscarinic signaling impairment would be uniquely predictive of schizophrenic syndrome. The grading of this signal deficit would correlate with non-deficit and deficit subtyping (**Figure 9**). In this figure schizoaffective disorder shares the same genetic risk profile as bipolar disorder and schizophrenia. The Maudsley twin study of non-hierarchically defined syndromes justifies this broader grouping (Cardno et al., 2002). Symbol M refers to muscarinic transduction. CACNA1C is a risk gene common to bipolar, schizophrenic and major depressive disorders (Green et al., 2010). It codes for a subunit of the L-type voltage-gated calcium channel.

The nomenclature is consistent with single or double allelic deletions, but can also be viewed as relative signaling deficit where other large effect CNVs that directly impact on elements within the muscarinic pathway are yet to be discovered. For example, M4 receptor transactivation of tyrosine kinases (Src) and thus Akt/ERK pathways is mediated by Gβγ subunits. Complicating matters, convergent muscarinic M1-type mediated ERK phosphorylation involves Src-dependent, PKC-independent transactivation of growth factor receptors or a PKC-dependent, Src-independent pathway, as demonstrated in salivary glands (Lin et al., 2008). This appears to be partly subtype determined, i.e., M1 or M3. The phenotypic expression of PLCB1 gene deletions depend on other factors and may not be necessary or sufficient to lead to the clinical psychotic episode. Epigenetic transformation of PLCB1 will interact with other susceptibility loci.

What then does a single case of PLCB1 deletion in the bipolar post-mortem cohort signify with respect to the proposed model of classification (Lo Vasco et al., 2013). Without detailed knowledge of the clinical history of the patient, which is not described in the paper, conclusions remain speculative. However, accepting the predictive value of a biological marker, a change in clinical diagnosis may be warranted: syndromes with single allele deletions would be classified as non-deficit and double deletions deficit schizophrenia. It requires replication in much larger population samples. This has outstanding implications for DSM classification by addressing the main criticism of the diagnostic system for a nosology lacking biological validity.

The role of PLCB1 and muscarinic signaling in neurodevelopment is likely to be rather complex. Although mutations in the PLCB1 gene could potentially be used as a marker differentiating a nosology of psychosis, the converse does not necessarily apply. This caveat refers to variance in phenotypic expression outside the psychotic domain. Thus, single case reports of deletions in chromosome 20p12.3 that disrupts PLCB1 and compound heterozygous gene mutations were implicated in intractable early infantile epilepsy (Kurian et al., 2010; Ngoh et al., 2014). In the latter study heterozygous deletion was detected in the mother and a separate mutation in the father. Both parents were apparently healthy, demonstrating incomplete penetrance of either disorder. There is a well-known overlap in genetic liability for epilepsy and schizophrenia. Obviously, M^-/-^ does not imply complete abrogation of function as some functional reserve or compensatory epigenetic changes will be consistent with non-lethality or absence of severe intellectual disability.

In these rare cases of infantile epilepsy the promotor sequence and first three coding exons are deleted. The probe for PLCB1 in the Lo Vasco study was 115.611 bp long spanning from exon 19 to 32. PLCB1 transcripts are alternatively spliced, but promotor deletion resulted in silencing of all the transcripts (Kurian et al., 2010). The limited data on schizophrenia suggests that deletions should allow functional transcripts of the mutated gene, but of significantly reduced potency. Of course, other factors might contribute to haplosufficiency, haploinsufficiency and incomplete dominance of the mutated allele. With adequate refinement of the probing technique and more data on PLCB1 mutations in schizophrenia, even a more accurate premorbid prediction might be possible.

Ultimately, coherent expression of emotions would depend on the integrity of PI3K/Akt or Raf/ERK pathways, but transduction oversensitivity of the same convergent pathways in theory, results in affective dysregulation. Clinical separation of an affective spectrum disorder from a diagnosis of schizophrenia with depressive features can often be tricky, so the value of a definitive genetic marker is obvious. There is also the potential of such a major genetic finding to help arbitrate when psychotic members of the same family are given different diagnoses.

## Conflict of Interest Statement

The author declares that the research was conducted in the absence of any commercial or financial relationships that could be construed as a potential conflict of interest.
